# Immune Reactivity to Raw and Processed Foods and Their Possible Contributions to Autoimmunity

**DOI:** 10.3390/foods14081357

**Published:** 2025-04-15

**Authors:** Aristo Vojdani, Elroy Vojdani, Carina Benzvi, Aaron Lerner

**Affiliations:** 1 Immunosciences Lab., Inc., Los Angeles, CA 90035, USA; 2 Cyrex Labs, LLC, Phoenix, AZ 85034, USA; 3 Regenera Medical, 11620 Wilshire Blvd., Ste. 470, Los Angeles, CA 90025, USA; evojdani@gmail.com; 4 Research Department, Chaim Sheba Medical Center, The Zabludowicz Research Center for Autoimmune Diseases, Tel Hashomer, Ramat Gan 52621, Israel; carina.ben.zvi@gmail.com (C.B.); aaronlerner1948@gmail.com (A.L.)

**Keywords:** immune reactivity, allergy, allergen, raw foods, processed foods, antibody-mediated, immunodominant epitopes, autoimmunity, cross-reactivity, antigenic mimicry

## Abstract

It is now known that diet or food is one of the environmental factors that can induce or contribute to autoimmunity. In a healthy person with a normal functioning immune system, food substances encounter no resistance and are allowed passage through the immune barriers without triggering immune reactivity. However, clinicians are becoming increasingly aware that modern food-processing methods can increase or decrease the immune reactivity of foods, including allergic reactions. Immune reactions to undigested food antigens could result in the production of IgE antibodies, which are involved in immediate immune reactivity, and in IgG and IgA antibodies, which are involved in delayed immune reactivity. Currently, measurements of these antibodies are generally only performed against antigens derived from raw foods. However, testing for food reactivity based only on raw food consumption is inaccurate because people eat both raw and cooked foods. Even home-cooked foods undergo different kinds of preparation or processing. Food processing can change the structure of raw food materials into secondary, tertiary, and quaternary structures that can have different reactive properties. This can affect the body’s normal oral tolerance of food, causing the immune system to mistakenly identify food as a harmful foreign substance and react to it immunologically, leading to food immune reactivity. This abnormal reaction to food molecules can lead to the production of antibodies against not just target food antigens but also the body’s own tissues, which can have significant implications in autoimmunity induction due to cross-reactivity and the other mechanisms discussed here. We hope that this present review will stimulate further research on the role of modified food antigens in the induction of autoimmunity based on some or all of the key points discussed in this review.

## 1. Introduction

Humankind has known about food allergies since time immemorial, even though they might not have always named it as such. Even ancient philosophers such as Hippocrates wrote that food could be responsible for adverse symptoms and even death, while Cato coined the famous statement that has been translated in modern times as “One man’s meat is another man’s poison” [1]. In 1902, Richet and Portier coined the term (translated) “anaphylaxis”, explaining it as the presence of a “toxogenin”, which, in combination with an offending substance in the patient’s blood, precipitated a systemic reaction [2]. In 1906, von Pirquet came up with the term “allergie” by combining the Greek words “allos” (altered) and “ergos” (reactivity) [3]. In 1963, Gell and Coombs devised the more expansive word “hypersensitivity,” with four main types, which, although somewhat modified, are still followed today: Type I—immediate hypersensitivity (IgE-mediated); Type II—cytotoxic hypersensitivity (IgG/IgM-mediated); Type III—immune complex-mediated hypersensitivity; and Type IV—delayed-type hypersensitivity (T cell-mediated) [4].

Unfortunately, it is not only the information and knowledge on food hypersensitivity that is growing, but the prevalence of the condition as well, which has increased significantly in both children and adults globally. In a 2022 review by Tanno and Demoly regarding the World Health Organization’s classification of food hypersensitivity (FH) [5], they stated that FH was a growing health problem worldwide that affects more than 10% of the general population, specifically 1 in 10 adults and 1 in 12 children. In the United States, new data (2021) from the Centers for Disease Control (CDC) report that 6.2% of US adults have a food allergy, while 5.8% of children are afflicted [6,7].

This trend in increased food immune reactivity is measured either by skin testing or by in vitro testing such as enzyme-linked immunosorbent assays (ELISAs) [8,9,10]. However, despite these advancements in our knowledge of food hypersensitivity, many false-negative and false-positive results still occur during allergy testing due to the lack of standardized food allergens [11,12,13]. The skin prick test, for one, although highly sensitive, often shows low specificity. This is largely due to the complexity involved in manufacturing food allergen extracts, which requires several steps, including

- Selection and identification of source materials;
- Grinding and defatting;
- Extraction under specific conditions;
- Antigenic purification;
- Sterilization;
- Product testing.

Variations in any of these steps can significantly impact the quality and allergenicity of the final food product. Notably, although extracts used for allergen immunotherapy in the United States are prepared from raw source materials, many foods are consumed in processed or cooked forms. Thermal and non-thermal industrial food processing prior to extraction can alter the allergenicity of certain common food allergens, potentially affecting test results [14].

Among the critical factors in food allergy testing, the extraction methodology and whether the food is raw or processed stand out as among the most crucial factors for enhancing specificity in both in vivo and in vitro food allergy tests [15]. This is particularly true when comparing allergens from raw versus processed foods.

The term “processed foods” refers to foods that have undergone alterations from their natural state through various processes such as cutting, adding ingredients, heating, pasteurization, canning, refrigeration, freezing, drying, and refining.

García et al. [15] developed a novel extraction method using disaggregating agents to reduce the levels of gliadin homopolymers and heteropolymers formed during heat processing of food. Disaggregating agents are substances, often small molecules or proteins, that can break down or dissolve protein aggregates, like amyloid plaques, which are implicated in diseases like Alzheimer’s and Parkinson’s [16]. Garcia’s method demonstrated a significant improvement in gliadin extraction from foods processed at temperatures exceeding 200 °C. In contrast, the conventional extraction method using 60% ethanol only extracted 14.4% of the gliadin from wheat flour, whereas the cocktail containing disaggregating agents extracted 95.5% [15].

Despite this and other reports showing variations in antibody reactivity to raw versus processed food antigens, most skin testing and antibody measurement for both immediate (IgE) and delayed food sensitivity (IgG, IgA, and immune complexes) are still performed using extracts prepared from raw foods [17,18,19,20]. This is despite Prausnitz first documenting a case of food allergy to cooked food back in 1921 [21].

## 2. Why Raw Versus Processed Foods Matters

Even though there is a documented rise in food allergies around the world [9,10], there is currently no reliable test method for its detection, possibly due to the unavailability of standardized allergens extracted from raw and processed foods. Processed foods and their ingredients are subjected to different treatments and conditions, especially the thermal kind, which may affect food proteins and peptides in a manner that may induce the masking or unmasking of antigenic and allergenic epitopes, thereby either reducing or enhancing the possibility of their immunodominant epitopes affecting the antigenicity of the offending foods. Neo-antigen or neoallergen formation has been known for more than four decades [22,23]. One of the most recent examples of a very frequently used food additive turning naïve protein to an immunogenic one is microbial transglutaminase [24,25,26,27].

This may partly explain why some people can have no reaction to a raw food or a raw-food ingredient but will react to the same food when it is processed. Studies have found neoallergens from pecans [23], wheat flour [28], roasted peanuts [29], lentils [30], almonds, cashew nuts and walnuts [31], soybean [32,33], shrimp, scallops, tuna, egg, apples, plums, milk, potatoes, and many others [9,34,35,36,37,38]. Thermal processing may be performed using dry heat (e.g., oven roasting, oil roasting, ohmic heating, and infra-red heating) or wet heat (e.g., boiling, steaming, microwave cooking, pressure cooking, autoclaving, extrusion, and blanching) [39]. The previously mentioned articles also showed that in a majority of cases, some of the technological processing treatments not only maintained the antigenicity and allergenicity of the foods, but also induced the introduction and modification of neo-antigens.

More recent studies have gone further to differentiate foods that have simply been home-cooked from foods that have been industrially processed. The latter have now been dubbed ultra-processed foods (UPFs), and are defined as foods that have undergone extensive industrial processing, typically involving multiple steps and the addition of ingredients not usually used in simple home cooking, such as high levels of food additives.

A 2024 publication in BMJ by Lane et al. found a direct association between exposure to UPFs and 32 health parameters spanning mortality, cancer, mental, respiratory, cardiovascular, gastrointestinal, and metabolic health outcomes [40]. The researchers concluded that human health would be improved by reducing dietary exposure to UPFs such as packaged snacks, carbonated soft drinks, instant noodles, and ready-made meals, to name a few.

This broad review of the range of diseases arising from UPF consumption is supported by other numerous studies that have a more specific or narrower view but which nonetheless provide valuable information.

For instance, Whelan et al. in 2024 found evidence for an association between UPFs and gut disease, including inflammatory bowel disease, colorectal cancer, and irritable bowel syndrome [41], reviewing the effects of emulsifiers, sweeteners, colors, microparticles, and nanoparticles on the diet–microbiome–intestinal trialogue.

A UK-based cohort study by Chang et al. in 2023 found indications that higher UPF consumption may have a role in increased overall and some site-specific cancers, especially ovarian cancer [42].

And in a mouse model in 2021, Snelson et al. demonstrated that long-term consumption of processed foods drives intestinal barrier permeability, chronic kidney disease, and diabetes [43]. Enhanced intestinal permeability to undigested proteins and peptides originated from modified foods is considered the gateway to autoimmunity [44].

### 2.1. Detection of IgE, IgG, and IgA Antibodies Against Raw and Modified Food Antigens

In a study we conducted in our lab in 2009 [17], we used 80 different sera: 40 with confirmed IgE, IgG, and IgA reactions to food, and 40 from normal controls. All 80 were tested for the presence of IgE-, IgG-, and IgA-specific antibodies against about 200 food items in raw and modified forms. Overall, we found higher levels of these antibodies against some of these foods in the group with known IgE reactions. After the application of sera from highly reactive individuals to modified food end-products and their base or raw ingredients, we found higher elevations in IgE, IgG, and IgA antibody levels against processed foods versus their raw food antigens in 9 serum samples from individuals with confirmed food allergies. For example, in apple extract versus apple cider, or wheat, egg, corn, and milk versus cake. Compared to sera from healthy controls, in a majority of the specimens with strong IgE reactions to food, this immune reaction against different modified food products was higher than the reaction against raw ingredients of the same food [17].

These findings confirm that food modification results in the generation of many new immunodominant proteins and peptides that are very hard to digest. The presentation of these new epitopes to the immune system may result in IgE, IgG, and IgA antibodies against these immunodominant proteins, peptides, or new epitopes. These antibodies would not be detected by any immunological assay if the food proteins and peptides used were extracted only from raw food.

**Figure 1:**
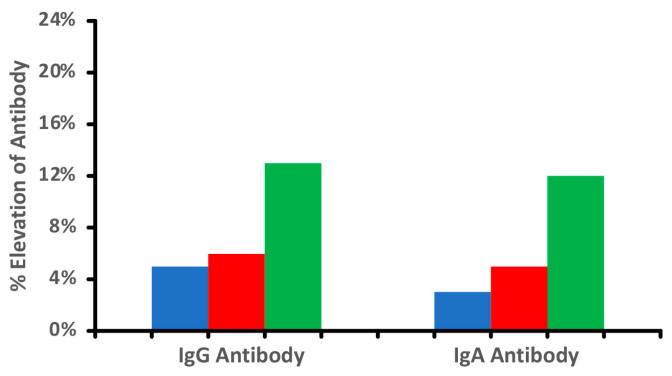
Percentage elevation in IgG and IgA antibodies against raw 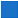, cooked 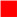, and tandoori chicken 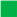 at a mean cutoff of 0.9 ELISA units. n = 40.

It is important to note that not all food modification is accomplished through heat. Foods can be subjected to freezing, drying, marination, chemicals, and various enzymes including microbial ones. The bright red color of chicken tandoori, for instance, may come as much from the artificial coloring known as Red 40 (Allura Red AC) as from red chili powder. Figure 1 shows the difference in antibody reactivity when comparing raw chicken, cooked chicken, and tandoori chicken. It is evident that more individuals reacted to the tandoori chicken, which may be due to the addition of chemical color and spices, causing the formation of neo-antigens and resulting in the production of more antibodies. Additionally, it is well documented that processed meat products are prepared with microbial transglutaminase, which was recently documented to induce autoimmune diseases [24,25,26,27]. The above shows that exposure of food to high temperatures, various food additives, or enzymes results in the generation of additional immunodominant proteins and peptides, as demonstrated in our aforementioned study [17].

Our data and the findings from other investigations further confirmed that food additives or thermal processing results in the generation of new immunodominant epitopes and the production of much higher antibody levels in some individuals [24,25,26,27,39,45,46,47].

### 2.2. Why Traditional Food Sensitivity Testing Falls Short

One major limitation of traditional food sensitivity testing is its lack of relevance to real-world, modern diets. Most widely used food antigen tests rely solely on raw food isolates. Yet, since humans first began cooking, diets have evolved to include mostly cooked and processed foods. Research has shown that cooking and processing alter the chemical and molecular structure of foods [47,48]. These changes affect individual food components and also interact when foods are combined during preparation, inducing post-translational modifications of the proteins. For example, a person might react to ketchup but not to raw tomatoes. However, if tested with traditional food sensitivity assays that only use raw tomato extracts, the person may test negative for a tomato allergy, leaving the actual sensitivity unresolved [23,24,25,26,27,28,29,30,31,32,36,39,40,41,42,43,44,45,46,47].

Due to these complex interactions, standard food sensitivity tests may not fully capture how a person’s immune system responds to food in daily life. The tests often ignore the differences introduced by cooking and processing, which may explain why some individuals continue to experience symptoms despite negative test results.

Moving beyond raw food isolates in testing and incorporating more real-world food preparations could significantly improve the accuracy of food sensitivity detection and assessments.

The awareness of the effect of food processing on food allergenicity continues to grow. In a 2018 review, Rao et al. already showed that processing peanuts by roasting or boiling made them more digestible and affected the binding of IgE antibodies to different degrees [48]. In 2021, Cuadrado et al.’s own review on nut allergenicity determined that the effect of food processing on the allergenicity of the nuts was variable, depending on the characteristics of the nut protein and the type and duration of treatment [49].

In 2025, Gonzalez et al. built upon Cuadrado’s findings on the variability of the effects of different types of food processing and proposed that by identifying the processes that reduced allergenicity, they could then be used to produce less-allergenic foods for treatment and diet liberation [50]. In their review, Gonzalez et al. cited studies that found that a majority of children allergic to milk and eggs were tolerant of these foods in “baked” form, that is, mixed in a batter and then baked. They also detailed the effects of other kinds of food processing, from pasteurization to canning to roasting and even peeling [50].

## 3. Why Immunodominant and Neo-Peptides Matter

Each food contains various proteins that, during digestion, break down into numerous peptides, which are introduced to the immune system. The immune response, however, does not target every peptide equally; instead, it focuses on a few dominant peptides, known as immunodominant peptides, in a process called immunodominance. This selectivity determines the specific peptides to which the immune system reacts, and under certain conditions, such as a lack of digestive enzymes or a leaky gut, this can often break down tolerance and lead to immune responses and antibody production. Immune tolerance is defined as the epitope-specific suppression of unnecessary humoral and cellular immune responses. A unique population of dendritic and regulatory T cells plays a crucial role in this process, interacting with dietary proteins and peptides to determine if the immune response should be more reactive (antibody production) or tolerant (lack of antibody production) [51]. In addition, dietary proteins undergo digestion by salivary and digestive enzymes, as well as by gastric acid. This process results in reduced immunogenicity of proteins by destroying the immunodominant or conformational peptides. Interestingly, human gut proteases fail to digest industrially processed cross-linked protein complexes. It appears that the very frequently used microbial transglutaminase cross-linker induces proteinase-resistant iso-peptide bonds [24,25,26,27]. Recently, manipulations of this enzyme were shown to increase its thermoresistance, further enhancing its potential harmful effects on human health [52,53,54,55].

## 4. The Formation of Neo-Antigens and Immunodominant Epitopes by Thermal and Other Modifications

The thermal and various industrial modifications of food result in protein denaturation, glycosylation, cross-linking, post translational modifications, and the formation of new antigens and peptides known as neo-antigens, which have an enhanced resistance to digestion even after entry into the small intestine [25,26,27,56,57].

This process generates immunodominant epitopes—key areas of a protein that the immune system recognizes and targets. These new epitopes can stimulate the production of antibodies, especially when compared to raw food antigens. In essence, cooked or processed foods may expose the immune system to altered peptide structures that provoke stronger immune responses.

For example, in gluten-containing foods, heat, other cooking methods, or food additives like bacterial enzymes can alter gluten protein structures, forming new immunodominant epitopes. While some individuals may react to specific proteins in gluten’s primary structure (like gliadin or glutenin), others may only react to the complex, combined structure that gluten forms during food preparation. This structural shift can lead to a more intense immune reaction, as seen in certain autoimmune and inflammatory responses [50,52,55].

**Figure 2:**
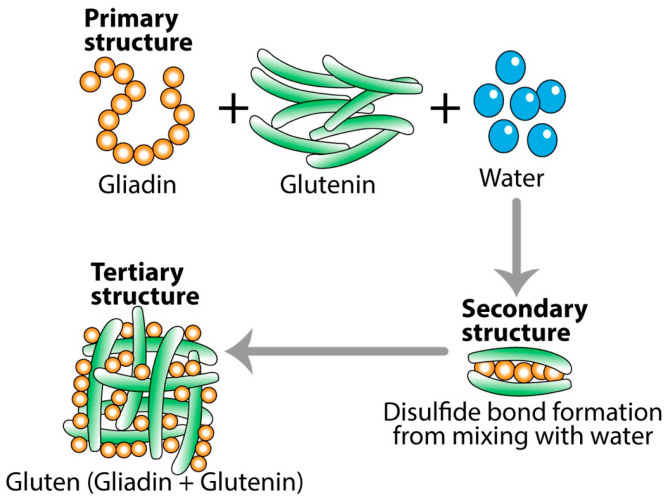
Transformation of gliadin and glutenin into gluten. One may react to the primary structure form of gliadin or glutenin, but not to the modified tertiary form of gluten. However, many may react to the tertiary structure of gluten, but not to either gliadin or glutenin.

One of the best examples of the effect of temperature on the digestibility of food in the digestive tract is what happens to gluten protein and celiac peptides during baking [57]. During dough formation, gliadin and glutenin combine into an elastic protein network called gluten macropolymer (GMP) (see Figure 2). When the raw flour is mixed with water, the primary structure of the combined gliadin, glutenin, and water is transformed into the secondary structure of raw pizza or bread dough. During baking, the GMP structure is further altered to a degree that affects the digestibility and immunogenic properties of gluten, initiating immune responses during celiac pathogenesis [57].

This highlights the fact that there are other mechanisms or factors aside from thermal modification that can form or affect neo-antigens and immunodominant epitopes. For instance, the processed food additive microbial transglutaminase can cross-link with gluten, thus turning the naïve gluten into an immunogenic molecule and producing neo-epitopes. This post translational modification is a key mechanism for molecular mimicry and autoimmunogenesis [47,50]. Some of these resulting neo-epitopes can increase immunogenicity, while others can decrease it.

This decrease in digestibility and increase in immunogenicity of proteins and peptides is not unique to gluten, and has been shown in many other foods, including rice [58] and soy [59].

**Figure 3:**
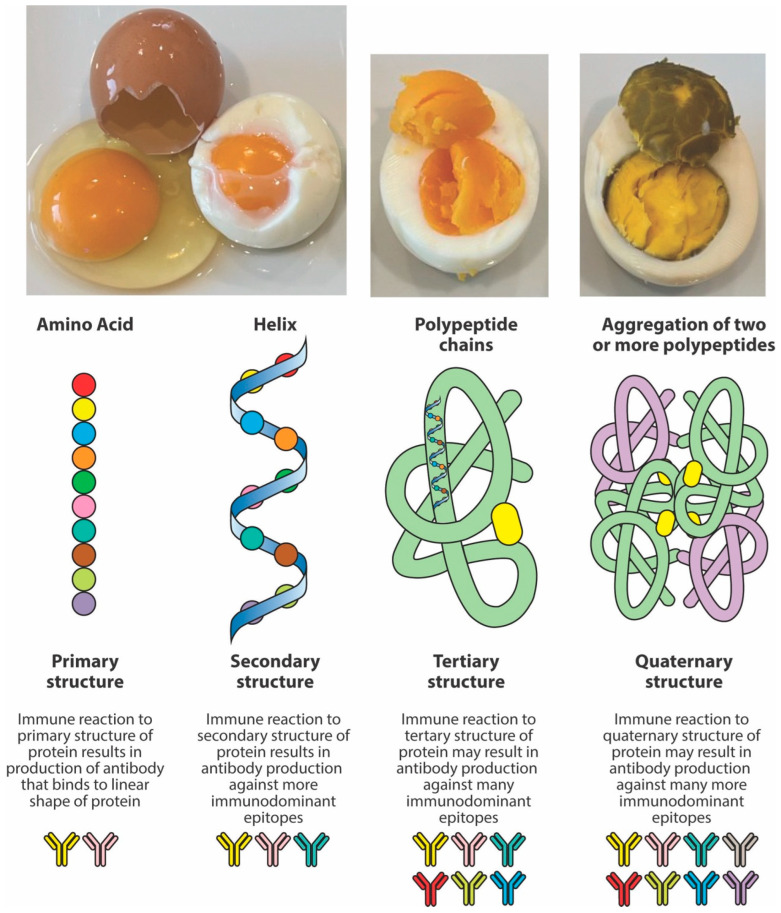
The effect of heat on peptide structure. Heat changes the structure of proteins and peptides from the original native chain of amino acids or primary structure to an alpha helix or secondary structure, and then to the more complex tertiary and quaternary structures. A protein has multiple immunodominant epitopes across the primary, secondary, tertiary, and quaternary structures, but generally, the tertiary and quaternary forms are likely to have the most immunodominant epitopes due to protein folding and their complex 3D conformation, exposing more potential binding sites for antibody reactions.

Thus, the structure adopted by peptides depends not only on the primary sequence of amino acids but also on conditions such as heat, solvent polarity, and method of sample preparation. In one study [60], it was shown that, under certain conditions, peptides may shift from a monomeric alpha helix to aggregated beta-sheet conformations or polypeptide chains. Continuous exposure to higher degrees of heat results in the aggregation of two or more peptides (see Figure 3). Consequently, it is possible that one may immunologically not react to the primary structure of a specific peptide but to the tertiary or quaternary structures of the same peptide’s polypeptide chains formed after exposure to high temperatures. Therefore, compared to the antigens or peptides from raw food proteins, the measurement of antibodies against peptides, particularly against their aggregated polypeptide chains, does matter.

**Figure 4:**
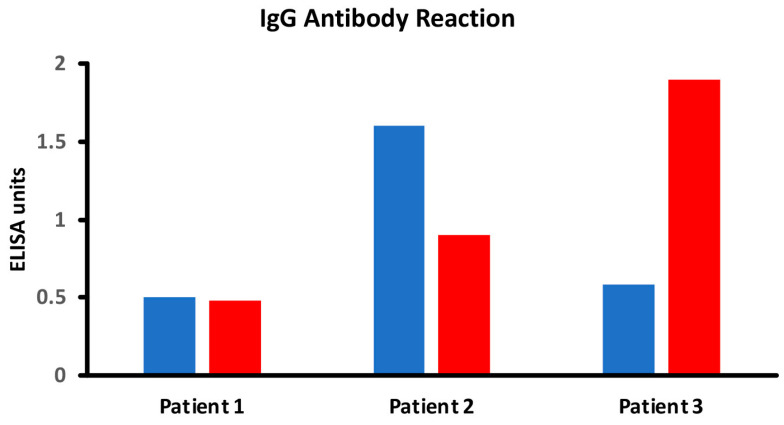
Different scenarios of raw 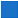 vs. cooked 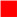 foods. Patient 1 shows no significant reaction to either raw or cooked egg. Patient 2 shows a significant reaction to raw egg but not cooked egg, while Patient 3 shows a significant reaction to cooked egg but not raw egg.

In the intestinal lumen, food proteins are digested into small or large peptides. If these peptides do not fully convert into amino acids, they can elicit both cellular- and antibody-mediated immune responses. Thermal modification, such as cooking or other food manipulations, often stabilizes these peptides into secondary, tertiary, and quaternary structures that resist enzymatic digestion. Thus, in comparison to raw foods, cooked foods are more likely to present many undigested immunodominant peptides to antigen-presenting cells and T cells, resulting in enhanced antibody production against these neo-peptides [17,56,57,58,59,60]. However, another possible scenario is that the denaturation of protein will hide some of these immunodominant epitopes compared to the more exposed ones of the linear protein molecule. In this case, the immune system may react to some epitopes on linear proteins, but not against cooked or modified food proteins, so that some individuals may produce more antibodies against the primary structure instead of the more complex structures of the modified food forms. Figure 4 shows examples of three different patients showing three different reaction scenarios involving raw vs. cooked foods. Many research studies have shown that individuals may not react against the primary structure or the native form of proteins or peptides, but may react to secondary, tertiary, and quaternary structures of the proteins and peptides due to denaturation, the Maillard reaction, and other factors [28,58,59,60].

## 5. Implications of Food in Autoimmunity

It is not just food allergy or food hypersensitivity that is increasing in prevalence around the globe. Incidences of autoimmunity are also increasing worldwide, and it would not be surprising if these two things were connected. In 2019, Gershteyn and Ferreira [61] came up with the word “immunodietica” to describe their data-driven approach to investigating interactions between diet and autoimmune disorders. Although their study dealt mainly with the link between the consumption of mammalian-origin proteins, such as pork and beef, and human autoimmunity, it laid the foundation for future studies on the impact of food antigens on the pathogenesis and severity of autoimmune diseases.

One of the significant risks with food antigens and their modified forms is cross-reactivity, where the immune system mistakenly identifies similar proteins in the body as harmful. This can lead to the production of antibodies that not only target food antigens, but also react with host tissues—a phenomenon seen in autoimmune conditions. Bioinformatic analyses have shown that autoreactive B cells and the antibodies produced by them mainly recognize non-linear or conformational epitopes that arise from protein folding into secondary and tertiary structures. Therefore, the detection of antibodies against linear epitopes of a protein is very different from antibodies against non-continuous regions of the amino acid chain that form the secondary and tertiary structures. Simply put, autoreactive B cells produce antibodies against neo-epitopes that are formed due to protein modification and occupy a precise three-dimensional conformational fit between food proteins and human tissue antigens, which play an important role in autoimmunity due to cross-reactivity [62,63,64,65,66,67,68]. This cross-reactivity can contribute to inflammation in the nervous system, potentially accelerating neurodegenerative processes. Additionally, in some studies, individuals with high levels of antibodies to processed food antigens also displayed elevated antibodies against myelin basic protein (MBP), a protein that plays a critical role in the nervous system, which is implicated in multiple sclerosis (MS) and other neurological conditions [67,68]. This suggests that dietary antigens may play a role in the progression of autoimmune diseases, particularly in individuals with underlying genetic predispositions.

Cross-reactivity between food antigens and human tissues may contribute to one of the major factors in autoimmunity: a breakdown in controlling food-related immune reactivity through the oral tolerance mechanism. Oral tolerance may fail due to many environmental factors that affect the human microbiome and the gut barriers, which may result in the production of antibodies against dietary antigens [69,70,71,72,73,74,75,76]. The presence of these antibodies in the blood and their reaction with target tissues may trigger or worsen autoimmune diseases, especially if the food antigens share structural similarities with human tissue components [27,77,78,79].

### Evidence for the Induction of Autoimmunity by Food Antigens Through Different Mechanisms

The following key points support the idea that food antigens contribute to autoimmunity.

1. There is molecular mimicry between food proteins/peptides and human tissues;
2. Polyclonal and monoclonal antibodies made against food antigens react with human autoantigens;
3. Antibodies made against human autoantigens react with various food antigens;
4. Affinity-purified antibodies from patients with a known autoimmune disease react with different food antigens;
5. Injection of food cross-reactive epitopes into animal models induces autoimmunity;
6. Food-specific antibodies and biomarkers of autoimmunity can be simultaneously measured using reliable blood tests.

**Key point 1: There is molecular mimicry between food proteins/peptides and human tissues**

**Figure 5:**
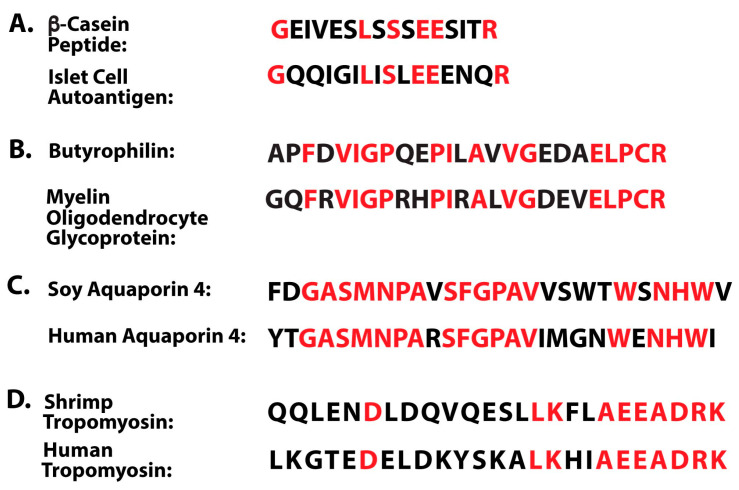
Amino acid sequence similarities between food antigens and human tissues. Sequence homologies are shown in red. (**A**) A β-casein peptide in cow’s milk and islet cell antigen. (**B**) Milk butyrophilin and myelin oligodendrocyte glycoprotein. (**C**) Human aquaporin-4, found in astrocytic processes within the brain and nervous system, has very significant similarities in amino acid sequence to four food aquaporins: corn, soybean, spinach, and tomato. Only soy is shown. (**D**) Shrimp tropomyosin shares similarities with human tropomyosin.

Molecular mimicry occurs when the amino acid (AA) sequence of a foreign substance shares similarity with the AA sequence of the body’s self-peptides to a degree that is sufficient to result in the cross-activation of autoreactive T and B cells and the production of antibodies that react with both the foreign and self-peptides. When the foreign protein or peptide comes from food, the result is molecular mimicry-based food immune reactivity. Normally, if undigested food proteins are somehow able to penetrate the gut barriers and enter the circulatory system, the body’s immune system will mount a response by producing defensive antibodies against those food proteins. However, if the food protein’s peptide sequence is sufficiently similar to the sequence of a human tissue, the immune system will produce antibodies against both the foreign matter and the self-tissue, resulting in autoreactivity and eventually autoimmune disease [79,80]. For instance, in one study on recent-onset insulin-dependent diabetes, a β-casein peptide in cow’s milk was shown to have antigenic similarity to islet cell autoantigen [81] (see Figure 5A). Other examples include milk butyrophilin (BTN) has significant homology with human myelin oligodendrocyte glycoprotein (MOG), as shown in Figure 5B, and food-sourced aquaporin-4 (AQP4), which is found in corn, soybean, spinach, tomato, and other plants, also shares sequence similarity with human AQP4, enough to cause significant cross-reactivity (see Figure 5C, which shows only soy). AQP4 has been implicated as having an important triggering role in the development of neuromyelitis optica (NMO), a neurologic disease characterized by inflammation of the optic nerve and spinal cord [82,83,84,85]. Finally, shrimp tropomyosin is similar enough to human epithelial tropomyosin for cross-reactivity to be possible (see Figure 5D), which may be why autoantibodies against human tropomyosin have been implicated as a causative agent in inflammatory bowel disorders [86,87,88,89,90].

In addition to these four examples, over the years, research has achieved great advances in the identification of peptide epitopes in food antigens that share similarities with the sequences of autoantigens involved in autoimmune diseases [91,92,93,94,95,96,97,98,99]. Some of these examples are summarized in Table 1.

**Key point 2: Polyclonal and monoclonal antibodies made against food antigens react with human autoantigens**

In addition to molecular mimicry between food antigens and human autoantigens, it is also very important to investigate the reaction of polyclonal and monoclonal antibodies made against food antigens with human autoantigens because it can directly indicate a potential for autoimmune disease. For example, when consuming gluten-containing foods, people with celiac and non-celiac gluten sensitivity may develop antibodies against these foods, which share homology with several human tissue antigens, such as tissue transglutaminase in the intestinal lining, thus causing damage to the lining and possibly other tissues as well [76,77,78,79,100,101,104]. Therefore, detecting food antibodies that strongly react with human autoantigens can be used to identify individuals at possible risk for developing autoimmune disease.

Examples of reactions of food-specific antibodies with human tissue antigens are shown in Table 2.

**Key point 3: Antibodies made against human autoantigens react with various food antigens**

In addition to molecular mimicry and the reaction of food antibodies with human tissue, the reactivity of tissue antibodies with different food proteins and peptides is also important because it further confirms the contribution of food proteins and peptides to autoimmunity [118,119,120,121].

It has been shown, for instance, that thyroid tissue is cross-reactive with many different antigens to the point of resulting in thyroid autoimmunity. Unfortunately, while there is a plethora of information about antigens of pathogenic organisms that are involved in thyroid autoimmunity through molecular mimicry [122], there is comparatively limited research regarding dietary proteins and how they affect thyroid function. In search of more helpful information, in one of our past studies [120], we used a monoclonal antibody made against triiodothyronine (T3), a thyroid hormone crucial for regulating metabolism, growth, and other bodily functions. We reacted the T3 antibody with antigens from 204 different foods. A total of 53 food proteins reacted with this antibody; of these 53 foods, 18 had weak reactions, 16 had moderate reactions, and 19 reacted very strongly.

Similarly, we took a monoclonal antibody against the thyroid hormone thyroxine (T4) and found 32 different food proteins that reacted with the antibody. Of the 32, 7 food proteins had weak reactions, 7 others had moderate reactions, and 18 had high immune reactivity. As for other thyroid antibodies, there was only one food each that reacted with a thyroglobulin antibody and deiodinase antibody, while none of the 204 foods reacted at all with the antibodies for thyroid-stimulating hormone receptor (TSHR) and thyroid-binding globulin [120]. However, since these reactions were obtained in a lab, we cannot definitively prove that the consumption of potentially reactive food proteins alone would be enough to elicit an inflammatory response against thyroid axis target sites. Other factors may have contributing roles, such as inactive digestive enzymes in the face of a breakdown in oral tolerance, enhanced gut permeability to large molecules, the composition of the gut microbiota, and a diet high in salt. These factors, either by themselves or in some combination thereof may increase the probability of developing immune reactivity to food and cross-reactivity with tissue antigens [78,120].

These and additional examples of tissue antibody reaction with food proteins are shown in Table 3.

**Key point 4: Affinity-purified antibody from patients with a known autoimmune disease react with different food antigens**

An alternate method of showing cross-reactivity between food antigens and host tissue is affinity purification of sera from patients with autoimmune disease, and then reacting the sera with tissue-specific target antigens. The subsequent reaction of disease-specific antibodies with food antigens identifies food antigens that cross-react with the human tissue antigens that are specific to the disease. For example, in one study, sera from lupus patients with high levels of antibodies against small nuclear ribonucleoprotein (Sm) were passed through a column containing the Sm antigen. The affinity-purified anti-Sm antibody was then tested against various food antigens using an ELISA. Among the many foods tested, the Sm antibody showed strong reactivity with soy, carrot, spinach, and corn antigens [123]. This finding suggests that these food antigens may contribute to the production of anti-Sm antibodies and could play a role in the pathogenesis of lupus.

In a similar study involving scleroderma patients, patient sera were purified to homogeneity through a column containing the Scl-70 antigen and then tested for reactivity to various food antigens. The Scl-70 affinity-purified antibody showed strong reactivity with plant DNA topoisomerase or enzymes found in corn, peas, spinach, and wheat germ agglutinin. The esophageal inflammation experienced by scleroderma patients may be linked to the exposure of the throat to these specific foods [124].

**Key point 5: Injection of food cross-reactive epitopes into animal models induces autoimmunity**

The design and use of animal models that can reproduce most clinical features of human disorders are critical not only for understanding the pathophysiology of autoimmune diseases, but are also critical for the development of new therapeutic modalities.

One of the ways to prove the involvement of certain cross-reactive proteins or peptides in autoimmunity is the administration of such autoantigens to animal models and the development of clinical symptomatology, T cells, or antibody responses associated with the specific autoimmune disease [125]. Induction of autoimmunity by tissue extract immunization was started as early as 1947 by Kabat et al. [126]. The introduction of homologous and heterologous brain tissue extracts prepared in complete Freund’s adjuvant (CFA) to rhesus monkeys were successful in the induction of experimental autoimmune encephalomyelitis (EAE). Since then, different tissue extracts have been applied to induce various autoimmune disorders in different animals [125,127].

Although animal models of autoimmune diseases, largely mice, have been and continue to be used to dissect their mechanisms as well as to test various therapeutic interventions [126,128]. Very little information is available in relation to the induction of autoimmunity by food proteins and peptides. As is shown in this review, dietary components that share homology with human tissues (food autoantigens) can trigger autoimmune responses if the immune system’s tolerance mechanisms fail [9,71].

One interesting approach to the induction of autoimmunity with food autoantigens was reported by Stefferl et al. in the year 2000 [80]. In their report, it was shown that molecular mimicry or cross-reactivity between myelin oligodendrocyte glycoprotein (MOG) and the special milk protein butyrophilin (BTN) can modulate the encephalitogenic T cell response to MOG in EAE. Furthermore, they demonstrated that the inflammatory response induced by the special BTN peptide 74–90 is responsible for the formation of scattered perivascular and meningeal macrophages and T cell responses that cross-react with MOG peptide. Moreover, they showed not only that adoptive transfer of MOG peptide 74–90-specific T cell lines mediated EAE, but treatment with homologous BTN peptide 74–90, injected or intranasally administered, could suppress disease activity induced by the MOG peptide. It was concluded that milk products such as BTN have the capacity to modulate pathogenic autoimmune responses due to cross-reactivity [80].

In addition to cross-reactivity, food components such as lectin and agglutinins may induce autoimmune disease due to their tendency to bind to human tissues and the gut microbiome. Acting as mitogens, lectins and agglutinins such as phytohemagglutinin and concanavalin-A, major components of raw beans, and pokeweed mitogen can activate both T cells and B cells, and thus may play a role in the induction of autoimmunities [128,129].

Dietary lectins are highly digestion-resistant and are able to cross the intestinal barriers and enter the circulation. From the blood, they may bind to and induce autoimmunity against different tissue antigens such as thyroid, pancreas, and joint components, inducing type 1 diabetes or rheumatoid arthritis [130,131,132]. In fact, in a rabbit model of arthritis, a single injection of lentil lectin into the knee joints induced the development of severe rheumatoid arthritis that was identified with lectin-specific antibodies and diagnosed with both acute and chronic phases that lasted up to one year [131,132].

Ovalbumin (OVA), a protein from egg whites, is a food protein that can only induce inflammatory arthritis after its raw form is mixed with CFA. This is achieved by OVA-specific T cells that may induce a breach in tolerance to endogenous antigens, leading to joint inflammation and autoimmunity [133].

Celiac disease (CD) is enteropathy of the small intestine induced by an a-gliadin 33-mer peptide or by dietary gluten in individuals who are DQ_2_/DQ_8_-positive. During the past two decades, several animal models, particularly those in non-human primates, were developed through repeated introductions of gluten or an a-gliadin 33-mer peptide. A better understanding of the complex cellular and molecular steps involved in the pathogenesis of CD can be gained by reading the publications of Costes et al., 2015, and Abadie et al., 2022 [134,135]. The availability of these models helps us understand the different pathways that are involved in the disease and aids in the development of new therapeutic targets beyond a gluten-free diet [134].

Unfortunately, this valuable information about the induction of autoimmunity by different food proteins and peptides is limited to milk, lectins/agglutinins, ovalbumin, and gluten. We still know nothing about the autoimmunogenic capacity of thousands of food proteins, and whether it is through molecular mimicry or other mechanisms. Furthermore, based on our own best knowledge and extensive literature searches, we have been unable to find any animal models that were developed to compare raw foods with processed food proteins and peptides.

For example, a mouse model of neuromyelitis optica (NMO) was developed through intradermal immunization with human AQP4 peptide 201–220, which induced paralysis in C57BL mice, similar to human NMO. Interestingly, the administration of an antibody made against IL-6 receptor to the mice was able to inhibit the induction of the clinical signs of the disease [136].

However, although it has already been shown that aquaporin from soy, corn, spinach, and tomato share more than 60% similarity with human aquaporin, so far, no attempt has been made to induce NMO using aquaporin from raw or processed foods.

**Key point 6: Food-specific antibodies and biomarkers of autoimmunity can be simultaneously measured using reliable blood tests**

Immune reactivity to food has been reported with increasing frequency in recent years, potentially driven by changes in the human microbiome caused by exposure to environmental factors associated with modern lifestyles [137]. These changes can affect the immune system’s ability to recognize food so that it may mistakenly identify it as a harmful foreign substance, resulting in food immune reactivity in some individuals.

This reaction to food antigens shows that the importance of accuracy in food immune reactivity testing cannot be overstated, especially for the measurement of IgG and IgA antibodies [18,138].

IgG- or IgA-mediated food immune reactivity does occur, and over the years, numerous blood tests have been developed for its detection. Many of them are poorly designed, and some do not measure antibodies [139,140,141,142,143,144]. For food immune reactivity testing to be accurate and reliable, the methodology for antibody detection must incorporate these four core principles: (1) pure antigens; (2) optimized antigen concentrations; (3) validation based on CLIA or other government agency requirements and standards; and (4) preparation of food antigens based on how the food is actually consumed in the real world [143,144]. Unfortunately, many labs do not follow these principles so the results from one lab can hardly be compared and correlated to the results of another lab [140,141,142,143,144]. Accurate lab testing for IgG and IgA antibodies against food antigens may be feasible in research labs that follow these principles, and the test findings could be used to design elimination diets for patients with autoimmune disorders [18,137,138]. Furthermore, the FDA has stated that “All allergenic extracts for the diagnosis of food allergy currently licensed by the FDA for distribution in the United States are non-standardized,” and this lack of uniformity makes accurate and reliable standardized testing for food immune reactivity a very difficult task [145].

To further correlate food protein antibodies with tissue-specific antibodies with which they may cross-react, simultaneous measurements of both food and tissue antibodies need to be performed with the same blood sample. We took such an approach in our lab in 2014 in relation to wheat and milk and their cross-reactive tissue epitopes [146]. Antigens of wheat, milk, and their components have been linked with neuroimmune disorders. We therefore measured the co-occurrence of their antibodies against various neural antigens. Sera from 400 donors were reacted with wheat and milk proteins, GAD-65, cerebellar, MBP, and MOG. The statistical analysis showed significant clustering when certain wheat and milk protein antibodies were cross-referenced with neural antibodies. About half of the sera with elevated antibodies against gliadin had significant reactions with GAD-65 and cerebellar peptides; about half of the sera with antibody elevation against α + β-casein and milk butyrophilin also showed antibody elevation against MBP and MOG. We concluded that in a subgroup of patients, cross-reactivity between α-gliadin and cerebellar peptides, and between milk butyrophilin and MOG peptides, could be responsible for the simultaneous detection of antibodies against these molecules in a small percentage of tested blood samples, which may have broader implications in the induction of neuroautoimmune reactivities [146].

**Figure 6:**
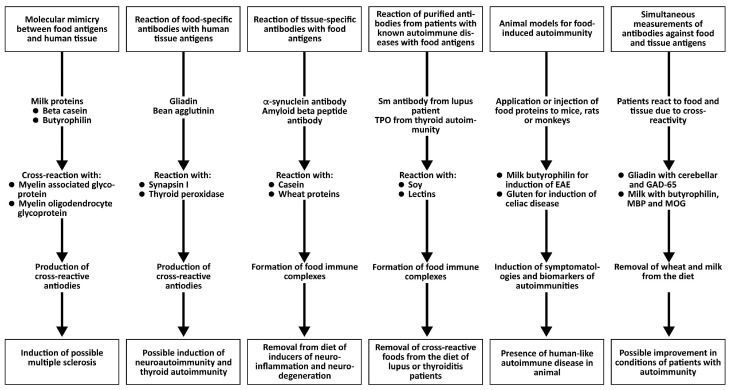
Different key points that support the concept of food-induced autoimmunity.

All the above key points provide evidence for the role of immune reactivity and cross-reactivity of foods with tissue antigens in autoimmunity (see Figure 6).

## 6. Correlation Between Food Modification, Formation of Advanced Glycation End Products (AGEs), and Protein Lipid Peroxidation and Their Contribution to Neuro-Autoimmunity

In our previous study [17] on the clinical significance of antibodies to modified food antigens in inflammation and autoimmunity, we tested an IgG antibody against AGE-human serum albumin (AGE-HSA) in individuals with low IgG antibody reactivity versus those with very high IgG antibody reactivity to modified food proteins. AGE stands for advanced glycation end-product. Cooking methods that involve high heat can lead to the formation of AGEs and other neo-antigens. Hemoglobin A_1_C and oxidized LDL are two good examples of AGEs. These molecules are highly immunogenic, and many significant neo-antigens found in processed foods are produced through the Maillard reaction [46], a chemical reaction that occurs when amino acids and reducing sugars in food are heated, causing the food to brown and develop flavor and aroma, especially in foods like meats, fried foods, foods with butter and oil, and processed items.

**Figure 7:**
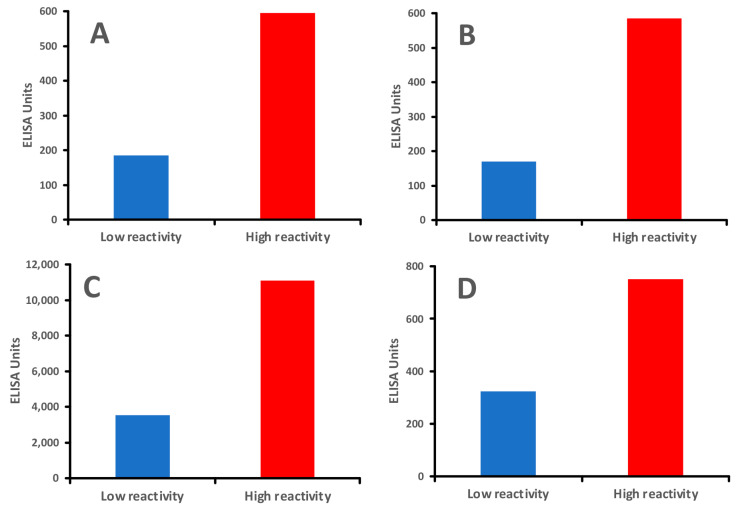
Individuals with greater reactivity to modified foods produced higher levels of IgG antibodies against human tissues than those with lower reactivity to modified foods. (**A**). IgG antibody against AGE-HSA mean of 8 individuals with low reactivity and 8 individuals with high reactivity to modified food antigens; (**B**). IgG antibody against AGE-Hemoglobin mean of 8 individuals with low reactivity and 8 individuals with high reactivity to modified food antigens; (**C**). IgG antibody against ox-LDL mean of 8 individuals with low reactivity and 8 individuals with high reactivity to modified food antigens; (**D**). IgG antibody against MBP mean of 8 individuals with low reactivity and 8 individuals with high reactivity to modified food antigens.

In comparison, healthy controls who showed immune reactivity with only a few processed food antigens produced moderate antibody levels against AGE-HSA, AGE-hemoglobin (AGE-Hb), and oxidized LDL (ox-LDL), whereas individuals with immune reactivity to many modified foods produced about three-fold higher levels of IgG antibodies against the same modified tissue antigens (Figure 7A–D).

Interestingly, the levels of antibodies against processed food antigens correlated directly with the levels of IgG against AGE-HSA, AGE-Hb, and ox-LDL.

These data suggest that the reaction of protein amino groups with sugar resulting in the formation of AGEs may be an important chemical pathway leading to distinct modification patterns in foods, which can stimulate the production of antibodies, leading to inflammatory responses. Studies have also linked AGEs to various chronic conditions, including diabetes, metabolic syndrome, and neurodegenerative diseases [147,148]. This connection suggests that ongoing exposure to modified food antigens, and the immune response they provoke, may contribute to systemic inflammation and age-related diseases.

Antibodies targeting AGEs have been shown to interact with other proteins, forming immune complexes that can deposit in tissues, further fueling inflammation and potentially leading to autoimmunity. Furthermore, antibody formations against ox-LDL and AGE-LDL are able to combine with circulating ox-LDL or AGE-modified antigens and form soluble immune complexes that may contribute to inflammation and autoimmunity [149,150].

Finally, since the earlier studies, it has been suggested that modifications to food antigens result in the formation of AGEs, and that AGEs play a significant role in neuronal stress, thus exacerbating aging and neurodegenerative processes in the brain. Much more recently, other researchers have also expounded on the connection between AGEs and neurodegenerative/neurocognitive disorders [151,152,153,154,155]. Interestingly, in another study [152], it was shown that autoantibodies from individuals with acute disseminated encephalomyelitis selectively bound to the folded myelin tetramer but not to its native form.

We also measured anti-MBP antibodies in patients that showed high levels of antibodies against processed food antigens (AGE-HSA, AGE-Hb, and ox-LDL). The results showed that the majority of samples with antibodies against modified proteins and lipoproteins also produced anti-MBP antibodies (see Figure 7D). The results of our study from 2009 [17] were further confirmed in 2021 in another study [156], which demonstrated that AGEs in food are triggering factors in the induction of self-reactivity against myelin antigens and in the production of antibodies against them in multiple sclerosis (MS). Altogether, these findings further support the idea that AGE formation by processed food antigens not only leads to the production of antibodies against dietary proteins and peptides, but could also result in the generation of antibodies against self-proteins such as myelin protein, possibly contributing to inflammation, autoimmunity, aging, diabetes, and neurodegeneration [147].

## 7. Conclusions: It Is Vital to Measure Antibodies Against Both Raw and Processed Food Antigens to Prevent or Treat Food Immune Reactivity and Autoimmunity

When primitive humans discovered fire, they found that cooking food makes it easier to eat and digest, and makes it tastier too. Fast forward to today, when we now have so many different ways of processing food. This only makes it more egregious that so many labs still persist in using mainly raw or unprocessed foods in their food reactivity testing, which limits its effectiveness in detecting sensitivities to processed foods. However, real-world diets include a range of processed foods that may contain modified proteins and antigens. Newer testing methods aim to address this gap by assessing immune responses to both raw and modified antigens. Testing for antibodies against these processed food antigens can provide a more accurate picture of an individual’s food sensitivities. Based on the reviewed literature and our findings, we propose that the determination of immune reactivity to food could be improved by measuring IgE, IgG, and IgA antibodies against both raw and processed food antigens. We have explained how foods with significant similarities to the structures or sequences of human tissues can lead to the production of antibodies that can target and attack not just the food antigens, but human tissues as well, potentially leading to autoimmunity. The removal of the identified immune-reactive processed foods from patients’ diets may contribute to the prevention of autoimmune and neurodegenerative disorders. Future research into the effects of food modification on immune responses may lead to more personalized approaches in dietary planning and food sensitivity testing. Recognizing the immune impact of processed foods and adopting a diet rich in minimally processed, anti-inflammatory foods may be key to managing immune-related health issues and improving overall well-being.

Thus, in addition to mimicry or cross-reactivity shared by raw and processed food proteins with human tissue, modification due to thermal control, glycation, peroxidation, and neo-antigen formation all play crucial roles in the induction of inflammation, autoimmunity, and neurodegenerative disorders.

It is now known that both genetics and environmental factors have roles in the induction of autoimmune diseases, with genetic predisposition accounting for about 30% of autoimmune disorders, and 70% being attributed to environmental factors. There may even be an interplay between the two, i.e., environmental factors may bring about autoimmune disease in those that are genetically predisposed to it [157]. Environmental factors are mainly divided into three groups: pathogens, toxic chemicals, and food. There is no end to the available literature on the associations between pathogens, toxic chemicals, and autoimmunity, but research into the connection between food, whether raw or processed, and autoimmune disorders is more limited.

We have presented six major key points that provide evidence and support for the concept of food-induced autoimmunity. As supporting evidence for the cross-reactivity between raw and processed foods, this review article does have limitations in that the lab work presented in it primarily stems from our own laboratory research [17,93,107,112,113,118], which may introduce bias. However, this should be viewed in the proper context, which is that there are not that many labs that do proper food antibody testing. Additional research by other laboratories is needed to confirm our results, and it is our hope that this review generates greater interest in studying the effects of raw versus processed foods in food sensitivity testing, and the role of raw and processed foods in autoimmunity, while following the same or similar principles recommended by CLIA and other government institutions.

## Figures and Tables

**Table 1:** Examples of molecular mimicry between food antigens and human tissue.

Food Proteins/Peptides	Cross-Reactive Tissue	Reference(s)
Wheat/gliadin Wheat germ agglutinin	Glutamic acid decarboxylase; hepatocytes; cerebellum; myelin basic protein; synapsin; asialoganglioside; amyloid beta; myelin-associated glycoprotein; microtubule-associated protein; thyroid peroxidase; alpha-enolase; tyrosinase; somatotropin; elastin; alpha-myosin	Vojdani et al. [91,93,94] Lerner and BenZvi [56,96] Natter et al. [95] Lerner et al. [96] Alaedini et al. [97]
Milk/casein/butyrophilin	Myelin oligodendrocyte glycoprotein; myelin-associated glycoprotein; islet cell antigen; zinc transporter 8; glutamic acid decarboxylase 65; retinal S antigen	Stefferl et al. [80] de Oliveira Andrade et al. [66] Guggenmos et al. [98] Riemekasten et al. [99] Monetini et al. [100] Chunder et al. [101] Wildner and Diedrichs- Möhring [102] Vieira Borba et al. [103]
Soy, corn, spinach, and tomato aquaporin 4, and serpin from legumes	Human aquaporin 4 found in the blood–brain barrier, astrocytes, cardiac myocytes, kidneys, lungs, stomach, skeletal muscle, salivary glands, and sensory organs	Vaishnav et al. [85] Kinoshita et al. [104] Lambert et al. [105] Shangzu et al. [106]
Corn and legume serpin	Aquaporin 4	Vaishnav et al. [85]
Shrimp and shrimp tropomyosin	Human tropomyosin	Das et al. [86,87] Vojdani and Vojdani [107]
Food pectins found in apple, orange, quince, berries, and grapefruit	Joints	Dai et al. [108,109]
Glycine-rich food proteins found in gelatin, meat, soy, chicken, eggs, cereals, beans, rice, and some fruits and vegetables	Procollagen; collagen; epidermal keratin	Lunardi et al. [110]
*Saccharomyces cerevisiae*	Complex glycolipids; alpha-synuclein	Newman et al. [111] Vojdani et al. [112]
Peanut proteins and lectins	Alpha-synuclein	Vojdani et al. [112]
Potato proteins	Alpha-synuclein	Vojdani et al. [112]

**Table 2:** Examples of food-specific antibody reactions with human tissue antigens.

Food Protein/Peptide Antibodies	Reactive Human Tissue	Reference(s)
Wheat/Gliadin	Synapsin I; cerebellar; hepatocytes; cytochrome P450; GAD-65; asialoganglioside; myelin basic protein; dopamine receptor; beta nerve growth factor; intrinsic factor; tyrosinase; thyroid peroxidase; alpha-enolase; e-cadherin; transglutaminase 2; elastin; alpha-myosin	Vojdani et al. [91,93,94] Alaedini et al. [97]
Wheat germ agglutinin	Myelin basic protein; zonulin; neurotropin; presenilin; tau protein; thyroid peroxidase; alpha-enolase; calmodulin; ovaries; insulin; islet cell antigen; alpha-myosin; acetylcholine receptor; dopamine receptor; neutrophil cytoplasmic antigen; placenta	Vojdani et al. [113] Kitano et al. [114] Debbage et al. [115] Jankovic et al. [116]
Bean and phytohemagglutinin	Intrinsic factor; calprotectin; hepatocytes; a+b tubulin; neurotrophin; tyrosinase; thyroid peroxidase; calmodulin; fibulin; platelet glycoprotein; alpha-myosin; dopamine receptor	Vojdani et al. [113]
Soy and soy bean agglutinin	Tyrosinase; thyroid peroxidase; alpha-myosin; alpha-enolase; intrinsic factor; asialoganglioside; aquaporin 4	Vojdani et al. [107,112,113]
Peanut and peanut agglutinin	Enteric nerve; cerebellum; tropomyosin; presenilin; ovaries; e-cadherin; insulin; islet cell antigen; dopamine receptor; amyloid beta peptide; glutamate receptor	Vojdani et al. [94,107,113]
Microbial transglutaminase	Mitochondrial M_2_ protein; extractable nuclear antigen; nuclear antigen; somatostatin; S-100B; alpha-myosin; transglutaminase 2; somatotropin; thyroid peroxidase	Lerner et al. [117]
Milk	Neutrophil cytoplasmic antigen; hepatocytes; thyroid peroxidase; alpha-enolase; somatotropin, myelin-associated glycoprotein	Vojdani [94] Chunder et al. [101]
Corn	Neurotrophin; intrinsic factor; alpha-myosin; thyroid peroxidase; tyrosinase; neutrophil cytoplasmic antigen; DPP-IV; somatotropin; aquaporin 4; myelin basic protein	Vojdani [94]
Egg	Zonulin; calprotectin; DPP-IV; alpha-enolase; lysozyme; myelin basic protein; asialoganglioside; aquaporin 4; platelet glycoprotein; fibulin; ovaries; insulin; islet cell antigen; alpha-myosin; dopamine receptor; acetylcholine receptor; amyloid beta peptide; brain-derived neurotrophic factor	Vojdani [94]

**Table 3:** Examples of tissue-specific antibodies that react with various food antigens.

Tissue Antibodies	Reactive Food	Reference(s)
Alpha synuclein	Casein; casomorphin; yeast; sorghum; onion; rice protein; bean agglutinin; pea lectin; lentil lectin; wheat germ agglutinin; soybean agglutinin; soy sauce; soy protein; tofu; potato; peanut; peanut butter; peanut agglutinin; macadamia nut; cashew; walnut; bell pepper; broccoli; lettuce; spinach; radish; celery; olive; latex hevein; grapefruit; bromelain; watermelon; shrimp; shrimp tropomyosin	Vojdani et al. [112,113]
Dopamine-1 receptor	Mint; eggplant; thyme; basil; cilantro; fish; wine; cherry; latex hevein; seaweed; cabbage; bell pepper; walnut; sesame albumin; chia seed; cashew vicilin; soybean oleosin; lentil lectin; rice protein	Vojdani and Turnpaugh [118]
Dopamine-2 receptor	Milk; cheese; wheat; peanut; peanut butter; walnut; seaweed; cherry; tuna; basil; mint; thyme	Vojdani and Turnpaugh [118]
Asialoganglioside GM_1_	Soy sauce; almond; chia seed; mustard seed; sunflower seed; asparagus; bell pepper; celery; chili pepper; eggplant; radish; spinach; cherry; date; wine; kiwi; orange; peach; basil; cilantro; parsley; thyme; cinnamon; paprika	Vojdani and Turnpaugh [118]
Amyloid beta^42^ peptide	Egg yolk; pea protein; pea lectin; raw and canned tuna; hazelnut; squid; cow’s milk; a+b casein; wheat proteins; alpha gliadin; wheat-alpha amylase; wheat globulin; CXCR3-binding gliadin	Vojdani and Vojdani [119]
Triiodothyronine (T3)	Roasted almond; amaranth; buckwheat; cow’s milk; milk chocolate; coffee; hemp; kamut; latex hevein; mustard seed; oat; egg yolk	Vojdani and Vojdani [119] Kharrazian et al. [120]
Thyroxine (T4)	Roasted almond; almond; Brussel sprout; Brazil nut; roasted cashew; cashew vicilin; macadamia nut; cooked clam; gelatin; mustard seed; oat; egg yolk	Vojdani and Vojdani [119] Kharrazian et al. [120]
Thyroid peroxidase	Pea lectin; lentil lectin; mushroom; okra; seaweed; apricot; avocado (raw and cooked); banana; lemon and lime; orange juice; cod; wheat germ agglutinin; cooked chicken; buckwheat; amaranth; quinoa; soy; potato	Vojdani and Vojdani [119] Kharrazian et al. [120]
Glutamic acid decarboxylase (GAD-65)	Buckwheat; amaranth; rice; corn; yeast; potato; quinoa; oat	Vojdani and Vojdani [119] Kharrazian et al. [120]
Zinc transporter-8 (ZnT8)	Seaweed; cooked lentil; pea protein; cooked pea; wheat; soybean oleosin; roasted peanut; tilapia; guar gum	Vojdani and Vojdani [119] Kharrazian et al. [120]
Islet cell antigen 2 (IA2)	Seaweed; pea lectin; bell pepper; zucchini; spinach; apricot; rice; garlic; guar gum	Vojdani and Vojdani [119] Kharrazian et al. [120]
Insulin receptor alpha	Milk butyrophilin; potato; amaranth; quinoa; tapioca	Vojdani and Vojdani [119] Kharrazian et al. [120]

## Data Availability

No new data were created or analyzed in this study. Data sharing is not applicable to this article.

## References

[1] Bahna S.L. (2024). History of food allergy and where we are today. World Allergy Organ. J..

[2] Portier P., Richet C. (1902). De l’action anaphylactique de certain venin. C. R. Seances Soc. Biol..

[3] Huber B. (2006). 100 years of allergy: Clemens von Pirquet—His idea of allergy and its imminent cconcept of disease. Wien. Klin. Wochenschr..

[4] Uzzaman A., Cho S.H. (2012). Chapter 28: Classification of hypersensitivity reactions. Allergy Asthma Proc..

[5] Tanno L.K., Demoly P. (2022). Food allergy in the World Health Organization’s international classification of diseases (ICD)-11. Pediatr. Allergy Immunol..

[6] Ng A.E., Boersma P. (2023). Diagnosed allergic conditions in adults: United States, 2021. NCHS Data Brief.

[7] Zablotsky B., Black L.I., Akinbami L.J. (2023). Diagnosed allergic conditions in children aged 0–17 years: United States, 2021. NCHS Data Brief.

[8] Mustilo P., Martin B.L., Oppenheimer J., Nelson M.R. (2019). Allergen immunotherapy extraction preparation instructional guide. Am. Coll. Allergy Asthma Immunol..

[9] Malsagova K., Stepanov A., Sinitsyna A.A., Izotov A., Klyuchnikov M.S., Kopylov A.T., Kaysheva A.L. (2021). Determination of specific IgG to identify possible food intolerance in athletes using ELISA. Data.

[10] Boyce J.A., Assa’ad A., Burks A.W., Jones S.M., Sampson H.A., Wood R.A., Plaut M., Cooper S.F., Fenton M.J., Arshad S.H. (2011). Guidelines for the diagnosis and management of food allergy in the United States; summary of the NIAID-sponsored expert panel report. Nutr. Res..

[11] Schoos A.M., Bullens D., Chawes B.L., Costa J., De Vlieger L., DunnGalvin A., Epstein M.M., Garssen J., Hilger C., Knipping K. (2020). Immunological outcomes of allergen-specific immunotherapy in food allergy. Front. Immunol..

[12] Abu El-Enin M.A., El-Din E.M.R.S., Abdelwahab H.W., El-Maksoud A.A., El-Aziz A.M.A., Shaaban M.I., Attia A.N., Aboukamar W.A., Mohei-Aldin S., Belal F. (2022). Preparation of chemically stable allergen-specific sublingual immunotherapy from Egyptian allergens. J. Clin. Lab. Anal..

[13] Terlouw S., van Boven F.E., Zonneveld M.B.-V., de Graaf-in ‘t Veld C., van Splunter M.E., van Daele P.L.A., van Maaren M.S., Schreurs M.W.J., de Jong N.W. (2022). Homemade food allergen extracts for use in skin prick tests in the diagnosis of IgE-mediated food allergy: A good alternative in the absence of commercially available extracts?. Nutrients.

[14] David N.A., Penumarti A., Slater J.E., Lockey R.F., Ledford D.K. (2020). Manufacturing food extracts. Allergens and Allergen Immunotherapy.

[15] García E., Llorente M., Hernando A., Kieffer R., Wieser H., Méndez E. (2005). Development of a general procedure for complete extraction of gliadins for heat processed and unheated foods. Eur. J. Gastroenterol. Hepatol..

[16] Kim J., Um H., Kim N.H., Kim D. (2023). Potential Alzheimer’s disease therapeutic nano-platform: Discovery of amyloid-beta plaque disaggregating agent and brain-targeted delivery system using porous silicon nanoparticle. Bioact. Mater..

[17] Vojdani A. (2009). Detection of IgE, IgG, IgA, and IgM antibodies against raw and processed food antigens. Nutr. Metab..

[18] Nowak-Wegrzyn A., Katz Y., Mehr S.S., Koletzko S. (2015). Non-IgE-mediated gastrointestinal food allergy. J. Allergy Clin. Immunol..

[19] Hill D.A., Grundmeier R.W., Ram G., Spergel J.M. (2016). The epidemiologic characteristics of healthcare provider-diagnosed eczema, asthma, allergic rhinitis, and food allergy in children: A retrospective cohort study. BMC Pediatr..

[20] Prescott S.L., Pawankar R., Allen K.J., Campbell D.E., Sinn J.K., Fiocchi A., Ebisawa M., Sampson H.A., Beyer K., Lee B.-W. (2013). A global survey of changing patterns of food allergy burden in children. World Allergy Organ. J..

[21] Prausnitz C., Küstner H. (1921). Studies on supersensitivity. Cent. Für Bakteriol..

[22] Spies J.R. (1974). Allergens. J. Agric. Food Chem..

[23] Malanin K., Lundberg M., Johansson S.G. (1995). Anaphylactic reaction caused by neoallergens in heated pecan nut. Allergy.

[24] Amirdivani S., Khorshidian N., Fidelis M., Granata D., Koushki M.R., Mohamadi M., Khostinat K., Mortazavian A.M. (2018). Effects of transglutaminase on health properties of food products. Curr. Opin. Food Sci..

[25] Bauer A., Rosiek P., Bauer T. (2025). Microbial transglutaminase—The food additive, a potential inducing factor in primary biliary cholangitis. Molecules.

[26] Hong G.P., Xiong Y.L. (2012). Microbial transglutaminase-induced structural and rheological changes of cationic and anionic myofibrillar properties. Meat Sci..

[27] Lerner A., Benzvi C., Vojdani A. (2024). The frequently used industrial food process additive, microbial transglutaminase: Boon or bane. Nutr Rev..

[28] Leduc V., Moneret-Vautrin D.A., Guerin L., Morisset M., Kanny G. (2003). Anaphylaxis to wheat isolates: Immunochemical study of a case proved by means of double-blind, placebo-controlled food challenge. J. Allergy Clin. Immunol..

[29] Maleki S.J., Chung S., Champagne E., Raufman J.P. (2000). The effects of roasting on the allergenic properties of peanuts. J. Allergy Clin. Immunol..

[30] Sandin D.I., San Ireneo M.M., Fernandez-Caldas E.F., Lebreo E.A., Borrego T.L. (1999). Specific IgE determinations to crude and boiled lentil (*Lens culinaris*) extracts in lentil-sensitive children and controls. Allergy.

[31] Su M., Venkatachalam M.V., Teuber S.S., Roux K.H., Sathe S.K. (2004). Impact of irradiation and thermal processing on the antigenicity of almond, cashew nut, and walnut proteins. J. Sci. Food Agric..

[32] Codina R., Oehling A.G., Lockey R.F. (1998). Neoallergens in heated soybean hull. Int. Arch. Allergy Immunol..

[33] Hefle S.L., Lambrecht O.M., Nordlee J.A. (2005). Soy sauce retains allergenicity through the fermentation production process. J. Allergy Clin. Immunol..

[34] Pastorello E.A., Ortolani C., Farioli L., Pravettoni V., Ispano M., Borga A., Bengtsson A., Incorvaia C., Berti C., Zanussi C. (1994). Allergenic cross-reactivity among peach, apricot, plum, and cherry in patients with oral allergy syndrome: An in vivo and in vitro study. J. Allergy Clin. Immunol..

[35] Sanchez C., Fremont S. (2003). Consequences of heat treatment and processing of food on the structure and allergenicity of component proteins. Rev. Fr. Allergol. Immunol. Clin..

[36] Sathe S.K., Teuber S.S., Roux K.H. (2005). Effects of food processing on the stability of food allergens. Biotechnol. Adv..

[37] Bock A.U., Atkins F.M. (1990). Patterns of food hypersensity during sixteen years of double-blind placebo-controlled food challenges. J. Pediatr..

[38] Rosen J.P., Selcow E., Mendelcon L.M., Grodofsky M.P., Factor J.M., Sampson H.A. (1994). Skin testing with natural foods in patients suspected of having food allergies: Is it a necessity?. J. Allergy Clin. Immunol..

[39] Davis P.J., Williams S.C. (1998). Protein modification by thermal processing. Allergy.

[40] Lane M.M., Gamage E., Du S., Ashtree D.N., McGuinness A.J., Gauci S., Baker P., Lawrence M., Rebholz C.M., Srour B. (2024). Ultra-processed food exposure and adverse health outcomes: Umbrella review of epidemiological meta-analyses. BMJ.

[41] Whelan K., Bancil A.S., Lindsay J.O., Chassaing B. (2024). Ultra-processed foods and food additives in gut health and disease. Nat. Rev. Gastroenterol. Hepatol..

[42] Chang K., Gunter M.J., Rauber F., Levy R.B., Huybrechts I., Kliemann N., Millett C., Vamos E.P. (2023). Ultra-processed food consumption, cancer risk and cancer mortality: A large-scale prospective analysis within the UK Biobank. eClinicalMedicine.

[43] Snelson M., Tan S.M., Clarke R.E., de Pasquale C., Thallas-Bonke V., Nguyen T.-V., Penfold S.A., Harcourt B.E., Sourris K.C., Lindblom R.S. (2021). Processed foods drive intestinal barrier permeability and microvascular diseases. Sci. Adv..

[44] Yazici D., Ogulur I., Pat Y., Babayev H., Barletta E., Ardicli S., Imam M.B., Huang M., Koch J., Li M. (2023). The epithelial barrier: The gateway to allergic, autoimmune, and metabolic diseases and chronic neuropsychiatric conditions. Semin. Immunol..

[45] Sen M., Kopper R., Pons L., Abraham E.C., Burks A.W., Bannon G.A. (2002). Protein structure plays a critical role in peanut allergen stability. J. Immunol..

[46] Davis P.J., Smales C.M., James D.C. (2001). How can thermal processing modify the antigenicity of proteins?. Allergy.

[47] Lerner A., Aminov R., Matthias T. (2016). Dysbiosis may trigger autoimmune diseases via inappropriate posttranslational modification of host proteins. Front. Microbiol..

[48] Rao H., Tian Y., Fu W., Xue W. (2018). In vitro digestibility and immunoreactivity of thermally processed peanut. Food Agri. Immunol..

[49] Cuadrado C., Sanchiz A., Linacero R. (2021). Nut allergenicity: Effect of food processing. Allergies.

[50] Gonzalez P.M., Cassin A.M., Durban R., Upton J.E.M. (2025). Effects of food processing on allergenicity. Curr. Allewrgy Asthma Rep..

[51] Vickery B.P., Scurlock A.M., Jones S.M., Burks A.W. (2011). Mechanisms of immune tolerance relevant to food allergy. J. Allergy Clin. Immunol..

[52] Lerner A., Matthias T. (2020). Processed food additive microbial transglutaminase and its cross-linked gliadin complexes are potential public health concerns in celiac disease. Internat. J. Mol. Sci..

[53] Böhme B., Moritz B., Wendler J., Hertel T.C., Ihling C., Brandt W., Pietzsch M. (2020). Enzymatic activity and thermoresistance of improved microbial transglutaminase variants. Amino Acids..

[54] Lerner A., Ramesh A., Matthias T. (2020). The temperature and pH repertoire of the transglutaminase family is expanding. FEBS Open Bio..

[55] Untersmayr E., Jensen-Jarolim E. (2008). The role of protein digestibility and antiacid on food allergy outcomes. J. Allergy Clin. Immunol..

[56] Lerner A., Benzvi C., Vojdani A. (2024). The potential harmful effects of genetically engineered microorganisms (GEMs) on the intestinal microbiome and public health. Microorganisms.

[57] Ogilvie O., Roberts S., Sutton K., Gerrard J., Larsen N., Domigan L. (2021). The effect of baking time and temperature on gluten protein structure and celiac peptide digestibility. Food Res Int..

[58] Liu K., Zheng J., Chen F. (2019). Effect of domestic cooking on rice protein digestibility. Food Sci. Nutr..

[59] Lan X., Liu P., Xia S., Jia C. (2010). Temperature effect on the non-volatile compounds of Maillard reaction products derived from xylose-soybean peptide system: Further insights into thermal degradation and cross-linking. Food Chem..

[60] Cerpa R., Cohen F.E., Kuntz I. (1996). Conformational switching in designed peptides: The helix/sheet transition. Fold. Des..

[61] Gershteyn I., Ferreira L. (2019). Immunodietica: A data-driven approach to investigate interactions between diet and autoimmune disorders. J. Transl. Autoimmun..

[62] Mackay I.R., Rowley M.J. (2004). Autoimmune epitopes: Autoepitopes. Autoimmun. Rev..

[63] Kamath S.D., Bublin M., Kitamura K., Matsui T., Ito K., Lopata A.L. (2023). Cross-reactive epitopes and their role in food allergy. J. Allergy Clin. Immunol..

[64] Jarius S., Paul F., Franciotta D., Waters P., Zipp F., Hohlfeld R., Vincent A., Wildemann B. (2008). Mechanisms of disease: Aquaporin-4 antibodies in neuromyelitis optica. Nat. Clin. Pract. Neurol..

[65] Gershteyn I.M., Burov A.A., Miao B.Y., Morais V.H., Ferreira L.M.R. (2020). Immunodietica: Interrogating the role of diet in autoimmune disease. Intern. Immunol..

[66] Andrade L.J.d.O., de Oliveira G.C.M., de Oliveira L.C.M., Bittencourt A.M.V., Baumgarth Y. (2024). Decoding the relationship between cow’s milk proteins and development of type 1 diabetes mellitus. Arch. Endocrinol. Metab..

[67] Vojdani A., Mukherjee P.S., Berookhim J., Kharrazian D. (2015). Detection of antibodies against human and plant aquaporins in patients with multiple sclerosis. Autoimmune Dis..

[68] Brandtzaeg P.E. (2002). Current understanding of gastrointestinal immunoregulation and its relation to food allergy. Ann. N. Y. Acad. Sci..

[69] Brandtzaeg P. (1998). Development and basic mechanisms of human gut immune function. Nutr. Rev..

[70] Vojdani A. (2015). Oral tolerance and its relationship to food immune reactivities. Alt. Ther. Health Med..

[71] Resigno M. (2011). Dendritic cells in oral tolerance in the gut. Cell Microbiol..

[72] Wu X., Liu J., Xiao L., Lu A., Zhang G. (2017). Alterations of the gut microbiome in rheumatoid arthritis. Osteoarthr. Cartil..

[73] Macdougall J.D., Kim E.H. (2023). A clinical focus on oral tolerance in the development, prevention, and management of food allergy. Cell. Immunol..

[74] Fu Y., Lyu J., Wang S. (2023). The role of intestinal microbes on intestinal barrier function and host immunity from a metabolite perspective. Front. Immunol..

[75] Lerner A., Matthias T. (2015). Changes in intestinal tight junction permeability associated with industrial food additives explain the rising incidence of autoimmune disease. Autoimmun. Rev..

[76] Schulze M.B., Martínez-González M.A., Fung T.T., Lichtenstein A.H., Forouhi N.G. (2018). Food-based dietary patterns and chronic disease prevention. BMJ.

[77] Bonds R.S., Midoro-Horiuti T., Goldblum R. (2008). A structural basis for food allergy: The role of cross-reactivity. Curr. Opin. Allergy Clin. Immunol..

[78] Vojdani A., Gushgari L.R., Vojdani E. (2020). Interaction between food antigens and the immune system: Association with autoimmune disorders. Autoimmun. Rev..

[79] Vojdani A. (2015). Molecular mimicry as a mechanism for food immune reactivities and autoimmunity. Altern. Ther. Health Med..

[80] Stefferl A., Schubart A., Storch M., Amini A., Mather I., Lassman H., Linington C. (2000). Butyrophilin, a milk protein, modulates the encephalitogenic T cell response to myelin oligodendrocyte glycoprotein in experimental autoimmune encephalomyelitis. J. Immunol..

[81] Cavallo M.G., Fava D., Monetini L., Barone F., Pozzilli P. (1996). Cell-mediated immune response to beta casein in recent-onset insulin-dependent diabetes: Implications for disease pathogenesis. Lancet.

[82] Jarius S., Wildemann B. (2010). AQP4 antibodies in neuromyelitis optica: Diagnostic and pathogenetic relevance. Nat. Rev. Neurol..

[83] Kim S.H., Kim W., Li X., Jung I.J., Kim H. (2012). Clinical spectrum of CNS aquaporin-4 autoimmunity. Neurology.

[84] Bradl M., Lassmann D.H. (2008). Anti-aquaporin-4 antibodies in neuro-myelitis optica: How to prove their pathogenetic relevance?. Int. MS J..

[85] Vaishnav R.A., Liu R., Chapman J., Roberts A.M., Ye H., Rebolledo-Mendez J.D., Tabira T., Fitzpatrick A.H., Achiron A., Running M.P. (2013). Aquaporin-4 molecular mimicry and implications for neuromyelitis optica. J. Neuroimmunol..

[86] Das K.M., Bajpai M. (2008). Tropomyosins in human diseases: Ulcerative colitis. Adv. Exp. Med. Biol..

[87] Das K.M., das Gupta A., Mandal A., Geng X. (1993). Autoimmunity to cytoskeletal protein tropomyosin: A clue to pathogenic mechanisms for ulcerative colitis. J. Immunol..

[88] Mirza Z.K. (2006). Autoimmunity against human tropomyosin isoforms in ulcerative colitis: Localization of specific human tropomyosin isoforms in the intestine and extraintestinal organs. Inflamm. Bowel Dis..

[89] Ebert E.C., Geng X., Bajpai M., Pan Z., Tatar E., Das K.M. (2009). Antibody to tropomyosin isoform 5 and complement induce the lysis of colonocytes in ulcerative colitis. Am. J. Gastroenterol..

[90] Zhang X., Li J., Wang F., Ren Y., Wu S., Wu Y., Tang Y. (2025). The clinical significance and biological function of tropomyosin 3 in ulcerative colitis. Tissue Cell.

[91] Vojdani A., O’Bryan T., Green J., McCandless J., Woeller K., Vojdani E., Nourian A., Cooper E. (2004). Immune response to dietary proteins, gliadin and cerebellar peptides in children with autism. Nutr. Neurosci..

[92] Rojas M., Restrepo-Jimenez P., Monsalve D.M., Pacheco Nieva Y., Acosta Ampudia Y.Y., Ramírez Santana H.C., Leung P.S., Ansari A.A., Gershwin M.E., Anaya J.M. (2018). Molecular mimicry and autoimmunity. J. Autoimmun..

[93] Vojdani A., Tarash I. (2013). Cross-reaction between gliadin and different food and tissue antigens. Food Nutr. Sci..

[94] Vojdani A. (2020). Reaction of food-specific antibodies with different tissue antigens. Int. J. Food Sci. Technol..

[95] Natter S., Granditsch G., Reichel G.L., Baghestanian M., Valent P., Elfman L., Grönlund H., Kraft D., Valenta R. (2001). IgA cross-reactivity between a nuclear autoantigen and wheat proteins suggests molecular mimicry as a potential mechanism in celiac disease. Eur. J. Immunol..

[96] Lerner B., BenZvi C., Vojdani A. (2024). Gluten is a proinflammatory inducer of autoimmunity. J. Transl. Gastro..

[97] Alaedini A., Okamoto H., Briani C., Wollenberg K., Shill H.A., Bushara K.O., Sander H.W., Green P.H.R., Hallett M., Latov N. (2007). Immune cross-reactivity in celiac disease: Anti-gliadin antibodies bind to neuronal synapsin I. J. Immunol..

[98] Guggenmos J., Schubart A.S., Ogg S., Andersson M., Olsson T., Mather I.H., Linington C. (2004). Antibody cross-reactivity between myelin oligodendrocyte glycoprotein and the milk protein butyrophilin in multiple sclerosis. J. Immunol..

[99] Riemekasten G., Marell J., Hentschel C., Klein R., Burmester G.R., Schoessler W., Hiepe F. (2002). Casein is an essential cofactor in autoantibody reactivity directed against the C-terminal SmD1 peptide AA 83-119 in systemic lupus erythematosus. Immunobiology.

[100] Monetini L., Barone F., Stefanini L., Petrone A., Walk T., Jung G., Thorpe R., Pozzilli P., Cavallo M. (2003). Establishment of T cell lines to bovine beta-casein and beta-casein-derived epitopes in patients with type 1 diabetes. J. Endocrinol..

[101] Chunder R., Weier A., Mäurer H., Luber N., Enders M., Luber G., Heider T., Spitzer A., Tacke S., Becker-Gotot J. (2022). Antibody cross-reactivity between casein and myelin associated glycoprotein results in central nervous system demyelination. Proc. Natl. Acad. Sci. USA.

[102] Wildner G., Diedrichs-Möhring M. (2003). Autoimmune uveitis induced by molecular mimicry of peptides from rotavirus, bovine casein, and retinal S-antigen. Eur. J. Immunol..

[103] Borba V.V., Lerner A., Matthias T., Shoenfeld Y. (2020). Bovine milk proteins as a trigger for autoimmune disease: Myth or reality?. Int. J. Celiac Dis..

[104] Kinoshita M., Nakatsuji Y., Kimura T., Moriya M., Takata K., Okuno T., Kumanogoh A., Kajiyama K., Yoshikawa H., Sakoda S. (2010). Anti-aquaporin-4 antibody induces astrocytic cytotoxicity in the absence of CNS antigen-specific T cells. Biochem. Biophys. Res. Commun..

[105] Lambert J., Mejia S., Vojdani A. (2019). Plant and human aquaporins: Pathogenesis from gut to brain. Immunol. Res..

[106] Shangzu Z., Dingxiong X., ChengJun M., Yan C., Yangyang L., Zhiwei L., Ting Z., Zhiming M., Yiming Z., Liying Z. (2022). Aquaporins: Important players in cardiovascular pathophysiology. Pharmacol. Res..

[107] Vojdani A., Vojdani E., Vojdani A., Bautista J. (2019). Chapter 7: Molecular mechanism for food immune reaction and autoimmunity. Food-Associated Autoimmunities.

[108] Dai H., Dong H.-L., Gong F.-Y., Sun S.-L., Liu X.-Y., Li Z.-G., Xiong S.-D., Gao X.-M. (2014). Disease association and arthritogenic potential of circulating antibodies against the α-1,4-polygalacturonic acid moiety. J. Immunol..

[109] Dai H., Gao X.M. (2011). Elevated levels of serum antibodies against alpha-1,6-glucan in patients with systemic lupus erythematosus or rheumatoid arthritis. Protein Cell..

[110] Lunardi C., Nanni L., Tiso M., Mingari M.C., Bason C., Oliveri M., Keller B., Millo R., De Sandre G., Corrocher R. (2000). Glycine-rich cell wall proteins act as specific antigen targets in autoimmune and food allergy disorders. Int. Immunol..

[111] Newman H.A., Romeo M.J., Lewis S.E., Yan B.C., Orlean P., Levin D.E. (2005). Gpi19 he *Saccharomyces cerevisiae* homologue of mammalian PIG-P, is a subunit of the initial enzyme for glycosylphosphatidylinositol anchor biosynthesis. Eukaryot. Cell.

[112] Vojdani A., Lerner A., Vojdani E. (2021). Cross-reactivity and sequence homology between alpha-synuclein and food products: A step further for Parkinson’s disease synucleinopathy. Cells.

[113] Vojdani A., Afar D., Vojdani A. (2020). Reaction of lectin-specific antibody with human tissue: Possible contribution to autoimmunity. J. Immunol. Res..

[114] Kitano N., Taminato T., Ida T., Seno M., Seino Y., Matsukura S., Kuno S., Imura H. (1988). Detection of antibodies against wheat germ agglutinin bound glycoproteins on the islet cell membrane. Diabet. Med..

[115] Debbage P.L., Hanisch U.-K., Reisinger P.W.M., Lange W. (1993). Visualization of lectin-like proteins in human placenta by means of anti-plant lectin antibodies. Anat. Embryol..

[116] Jankovic M. (2002). Identification of human placental wheat germ agglutinin immunoreactive protein by mass spectrometry. Comp. Biochem. Physiol. Part C Toxicol. Pharmacol..

[117] Lerner A., BenZvi C., Vojdani A. (2023). Cross-reactivity and sequence similarity between microbial transglutaminase and human tissue antigens. Sci. Rep..

[118] Vojdani A., Turnpaugh C.C. (2020). Antibodies against Group A Streptococcus, dopamine receptors, and ganglioside GM1 cross-react with a variety of food antigens, potentially interfering with biomarkers for PANS and PANDAS. Biomark. Neuropsychiatry.

[119] Vojdani A., Vojdani E. (2018). Immunoreactivity of anti-amyloid beta-peptide-42 specific antibody with toxic chemicals and food antigens. J. Alz. Dis. Park..

[120] Kharrazian D., Herbert M., Vojdani A. (2017). Immunological reactivity using monoclonal and polyclonal antibodies of autoimmune thyroid target sites with dietary proteins. J. Thyroid Res..

[121] Kharrazian D., Herbert M., Vojdani A. (2017). Detection of islet cell immune reactivity with low glycemic index foods: Is this a concern for type 1 diabetes?. J. Diabet. Res..

[122] Cuan-Baltazar Y., Soto-Vega E. (2020). Microorganisms associated to thyroid autoimmunity. Autoimmun. Rev..

[123] Bullard-Dillard R., Chen J., Pelsue S., Dao V., Agris P.F. (1992). Anti-Sm autoantibodies of systemic lupus erythematosus cross-react with dietary plant proteins. Immunol. Investig..

[124] Agris P.F., Parks R., Bowman L., Guenther R.H., Kovacs S.A., Pelsue S. (1990). Plant DNA topoisomerase I is recognized and inhibited by human Scl-70 sera autoantibodies. Exp. Cell Res..

[125] Yu X., Petersen F. (2018). A methodological review of induced animal models of autoimmune diseases. Autoimmun. Rev..

[126] Kabat E.A., Wolf A., Bezer A.E. (1947). The rapid production of acute disseminated encephalomyelitis in Rhesus monkeys by injection of heterologous and homologous brain tissue with adjuvant. J. Exp. Med..

[127] Morel L. (2025). Animal models of autoimmunity: A relentless pursuit of accurate pre-clinical models. Autoimmunity.

[128] Sjölander A., Magnusson K.E., Latkovic S. (1984). The effect of concanavalin-A and wheat germ agglutinin on the ultrastructure and permeability of rat intestine. A possible model for an intestinal allergic reaction. Int. Arch. Allergy.

[129] Wilson A.B., King T.P., Clarke E.M.W., Pusztai A. (1980). Kidney bean (*Phaseolus vulgaris*) lectin-induced lesions in rat small intestine: 2 Microbiological studies. J. Comp. Pathol..

[130] Vojdani A. (2015). Lectins, agglutinins, and their roles in autoimmune reactivities. Altern. Ther. Health Med..

[131] Brauer R., Thoss K., Henzgen S., Waldman G., Bog-Hansen T.C., Driessche E. (1985). Lectin-induced arthritis of rabbit as a model of rheumatoid arthritis. Proceedings of the Sixth International Lectin Meeting.

[132] Parkkinen J. (1989). Aberrant lectin-binding activity of immunoglobulin G in serum from rheumatoid arthritis patients. Clin. Chem..

[133] Howson P., Shepard N., Mitchell N. (1986). The antigen-induced arthritis model: The relevance of the method of induction to its use as a model of human disease. J. Rheumatol..

[134] Costes L.M.M., Meresse B., Cerf-Bensussan N. (2015). The role of animal models in unraveling therapeutic targets in coeliac disease. Best Prac. Res. Clin. Gastroenterol..

[135] Abadie V., Khosla C., Jadri B. (2022). A mouse model of celiac disease. Curr. Protoc..

[136] Serizawa K., Miyake S., Katsura Y., Yorozu K., Kurasawa M., Tomizawa-Shinohara H., Yasuno H., Matsumoto Y. (2023). Intradermal AQP4 peptide immunization induces clinical features of neuromyelitis optica spectrum disorder in mice. J. Neuroimmunol..

[137] Wong G.W.K. (2024). Food allergies around the world. Front. Nutr..

[138] Abrams E. (2022). Food allergy: Symptoms and diagnosis. J. Food Allergy.

[139] Benson T.E., Arkins J.A. (1976). Cytotoxic testing for food allergy: Evaluation of reproducibility and correlation. J. Allergy Clin. Immunol..

[140] American Academy of Allergy (1981). Position statements—Controversial techniques. J. Allergy Clin. Immunol..

[141] Potter P.C., Mullineux J., Weinberg E.G., Haus M., Ireland P., Buys C., Motala C. (1992). The ALCAT test: Inappropriate in testing for food allergy in clinical practice. S. Afr. Med. J. Apr..

[142] Mullin G.E., Swift K.M., Lipski L., Turnbull L.K., Rampertab S.D. (2010). Testing for food reactions: The good, the bad, and the ugly. Nutr. Clin. Pract..

[143] Hodsdon W., Zwickey H. (2010). Reproducibility and reliability of two food allergy testing methods. Nat. Med. J..

[144] Vojdani A. (2015). The evolution of food immune reactivity testing: Why immunoglobulin G or immunoglobulin A antibody for food may not be reproducible from one lab to another. Altern. Ther. Health Med..

[145] U.S. Food & Drug Administration All Allergenic Extracts for Diagnosis of Food Allergy: FDA Safety Communication—FDA Requires Warning about Anaphylaxis Following False Negative Food Allergen Skin Test Results in the Prescribing Information. https://www.fda.gov/safety/medical-product-safety-information/all-allergenic-extracts-diagnosis-food-allergy-fda-safety-communication-fda-requires-warning-about-anaphylaxis-following-false-negative-food-allergen-skin-test-results.

[146] Vojdani A., Kharrazian D., Mukherjee P.S. (2013). The prevalence of antibodies against wheat and milk proteins in blood donors and their contribution to neuroimmune reactivities. Nutrients.

[147] Ramasamy R., Vannucci S.J., Yan S.S., Herold K., Yan S.F., Schmidt A.M. (2005). Advanced glycation end products and RAGE: A common thread in aging, diabetes, neurodegeneration, and inflammation. Glycobiology.

[148] Bengmark S. (2007). Advanced glycation and lipoxidation end products—Amplifiers of inflammation: The role of food. JPEN.

[149] Virella G., Lopes-Virella M.F. (2003). Lipoprotein autoantibodies: Measurement and significance. Clin. Dev. Immunol..

[150] Turk Z., Ljubic S., Turk N., Benko B. (2001). Detection of autoantibodies against glycated and oxidized food proteins in diabetic patients. Clin. Chim. Acta.

[151] Reddy V.P., Aryal P., Darkwan E.R. (2022). Advanced glycation end products in metabolic and neurodegenerative disorders. Microorganisms.

[152] Mahayana N.P.K., Yadmika N.P.W.P., Aryaweda M.D.W., Mahardana M.D.P., Mamangdean C.T., Dewi N.N.A., Wirawan C., Laksmidewi A.A.A.P. (2024). Decoying the enemy: Soluble receptor for advanced glycation end products and cognitive impairment in neurodegenerative diseases—A systemic review and meta-analysis. Egypt J. Neurol. Psychiatry Neurosurg..

[153] de Almeida J.K.A., Brech G.C., Luna N.M.S., Iborra R.T., Soares-Junior J.M., Baracat E.C., Greve J.M.D., Alonso A.C., Machado-Lima A. (2024). Advanced glycation end products consumption and the decline of functional capacity in patients with Parkinson’s disease: Cross-sectional study. Clinics.

[154] D’cunha N.M., Sergi D., Lane M.M., Naumovski N., Gamage E., Rajendran A., Kouvari M., Gauci S., Dissanayka T., Marx W. (2022). The effects of dietary advanced glycation end products on neurocognitive and mental disorders. Nutrients.

[155] Kothandan D., Singh D.S., Yerrakula G., Backkiyashree D., Pratibha N., Ramya A., Vg S.R., Keshavini S., Jagadheeshwari M., Dhivya Sr K. (2024). Advanced glycation end products-induced Alzheimer’s disease and its novel therapeutic approaches: A comprehensive review. Cureus.

[156] Polykretis P. (2021). Advanced glycation end products as potential triggering factors in self-reactivity against myelin antigens in multiple sclerosis. Med. Hypotheses.

[157] Selmi C., Lu Q., Humble M.C. (2012). Heritability versus the role of the environment in autoimmunity. J. Autoimmun..

